# Vascular calcification and cardiac function according to residual renal function in patients on hemodialysis with urination

**DOI:** 10.1371/journal.pone.0185296

**Published:** 2017-09-27

**Authors:** Dong Ho Shin, Young-Ki Lee, Jieun Oh, Jong-Woo Yoon, So Yon Rhee, Eun-Jung Kim, Jiwon Ryu, Ajin Cho, Hee Jung Jeon, Myung-Jin Choi, Jung-Woo Noh

**Affiliations:** 1 Department of Internal Medicine, Kangdong Sacred Heart Hospital, Hallym University College of Medicine, Seoul, Korea; 2 Department of Internal Medicine, Kangnam Sacred Heart Hospital, Hallym University College of Medicine, Seoul, Korea; 3 Department of Internal Medicine, Chuncheon Sacred Heart Hospital, Hallym University College of Medicine, Chuncheon, Gangwon-do, Korea; The University of Manchester, UNITED KINGDOM

## Abstract

**Background:**

Vascular calcification is common and may affect cardiac function in patients with end-stage renal disease (ESRD). However, little is known about the effect of residual renal function on vascular calcification and cardiac function in patients on hemodialysis.

**Methods:**

This study was conducted between January 2014 and January 2017. One hundred six patients with residual renal function on maintenance hemodialysis for 3 months were recruited. We used residual renal urea clearance (KRU) to measure residual renal function. First, abdominal aortic calcification score (AACS) and brachial-ankle pulse wave velocity (baPWV) were measured in patients on hemodialysis. Second, we performed echocardiography and investigated new cardiovascular events after study enrollment.

**Results:**

The median KRU was 0.9 (0.3–2.5) mL/min/1.73m^2^. AACS (4.0 [1.0–10.0] vs. 3.0 [0.0–8.0], *p* = 0.05) and baPWV (1836.1 ± 250.4 vs. 1676.8 ± 311.0 cm/s, *p* = 0.01) were significantly higher in patients with a KRU < 0.9 mL/min/1.73m^2^ than a KRU ≥ 0.9 mL/min/1.73m^2^. Log-KRU significantly negatively correlated with log-AACS (ß = -0.29, *p* = 0.002) and baPWV (ß = -0.19, P = 0.05) after factor adjustment. The proportion of left ventricular diastolic dysfunction was significantly higher in patients with a KRU < 0.9 mL/min/1.73m^2^ than with a KRU ≥ 0.9 mL/min/1.73m^2^ (67.9% vs. 49.1%, *p* = 0.05). Patients with a KRU < 0.9 mL/min/1.73m^2^ showed a higher tendency of cumulative cardiovascular events compared to those with a KRU ≥ 0.9 ml/min/1.73m^2^ (*P* = 0.08).

**Conclusions:**

Residual renal function was significantly associated with vascular calcification and left ventricular diastolic dysfunction in patients on hemodialysis.

## Introduction

Cardiovascular disease is the leading cause of death in patients with end-stage renal disease (ESRD) [1,2]. However, traditional cardiovascular risk factors do not fully explain the elevated mortality rates of cardiovascular disease seen in patients with ESRD. Vascular calcification is a common pathologic finding among patients with ESRD that has a variety of forms, including the deposition of calcium in the intimal and/or medial vessel layer [3,4]. Previous studies suggested that this change in vessels might be induced by multiple factors such as using calcium-based phosphate binder, calcium-phosphorous products, oxidative stress, inflammation, and erythropoietin stimulating agent (ESA) resistance [5,6]. Eventually, vascular calcification greatly contributes to the exceedingly high cardiovascular disease mortality in this population [7]. Vascular calcification is related to vascular stiffness. Therefore, cardiovascular assessments including vascular calcification and stiffness are important to predict cardiovascular mortality in patient with ESRD. Of note, ambulatory blood pressure monitoring (ABPM), pulse wave velocity (PWV), and plain radiography are known to predict the severity of vascular calcification [8–10]. Especially, increased vascular stiffness, as measured by PWV, is associated with increased cardiovascular and all-cause mortality in patients with ESRD [11].

Residual renal function may contribute to better anemia and volume control, lower inflammation degrees, malnutrition, and calcium-phosphorous products, and greater solute clearance [12–15]. Thus, these beneficial effects explained the lower overall and cardiovascular mortality in dialysis patients with residual renal function. In fact, the importance of residual renal function in cardiovascular disease has been confirmed in many studies related to peritoneal dialysis [12–16]. There are several observational studies about the importance of residual renal function in patients on hemodialysis [17–20]. However, in most studies of hemodialysis, residual renal function was defined according to urine output and end points were analyzed using hard outcomes such as cardiovascular mortality. Therefore, identification of factors related with cardiovascular events was needed to evaluate them based on residual renal function expressed as glomerular filtration rate (GFR). Although several studies demonstrated the role of residual renal function expressed as GFR in phosphate management, anemia control, and solute clearance in patients on hemodialysis [21, 22], little is known about vascular calcification and cardiac function according to residual renal function expressed as GFR in patients on hemodialysis. Here we hypothesis that residual renal function was associated with vascular calcification and cardiac function and it affected cardiovascular events. Therefore, we investigated the correlation between residual renal function expressed as GFR and vascular calcification in patients on hemodialysis and conducted echocardiography in those patients. Furthermore, new cardiovascular events were evaluated after study enrollment.

## Materials and methods

### Ethics statement

This study was performed in accordance with the Declaration of Helsinki and approved by the Institutional Review Board of Kangdong Sacred Heart Hospital, Kangnam Sacred Heart Hospital, and Chuncheon Sacred Heart Hospital (Refs. 2014-01-025, 2014-04-54, 2014–96). Written informed consent was obtained from all patients prior to enrollment.

### Patients

This study was conducted at three dialysis clinics in Kangdong Sacred Heart Hospital, Kangnam Sacred Heart Hospital, and Chuncheon Sacred Heart Hospital between January 2014 and January 2017. One hundred and twenty, 111, and 108 patients were on Hemodialysis therapy in Kangdong Sacred Heart Hospital, Kangnam Sacred Heart Hospital, and Chuncheon Sacred Heart Hospital between January 2014 and February 2015, respectively. The inclusion criteria were as follow: patients with age ≥ 18 years with urination, patients with hemodialysis duration of at least three months, and patients with hemodialysis prescription of three times a week for four hours each time. Exclusion criteria were as follow: patients conducting hemodialysis due to chronic rejection after kidney transplantation, patients planning to transfer other hemodialysis center, and patients failing to undergo measurement of PWV or echocardiography. At three hospitals, 131 patients were considered eligible for study inclusion in that period. Of the 131 enrolled patients, four patients conducted hemodialysis due to chronic rejection after kidney transplantation, 12 patients planed to transfer other hemodialysis center, and nine patients did not undergo the measurement of PWV (n = 4) or echocardiography (n = 5). Thus, 106 patients were included in this study.

### Data collection

Baseline characteristics, including demographic, clinical, and biochemical data were obtained from medical records at the time of PWV measurement. Mean interdialytic weight gain was calculated as the average of 10 values after the time of PWV measurement. Echocardiography, abdominal plain radiography, urine collection of interdialytic period, and ABPM were conducted within one week at the time of PWV measurement.

### Definition

Cardiovascular disease was defined as a history of coronary artery disease, arrhythmia, cerebrovascular disease, or peripheral vascular disease; Coronary artery disease was defined as a history of angioplasty, coronary artery bypass grafting, myocardial infarction, or angina; Cerebrovascular disease was defined as a previous history of transient ischemic attack, stroke, or carotid endarterectomy; and Peripheral artery disease was defined as a history of claudication, ischemic limb loss and/or ulceration, or a peripheral revascularization procedure. Cardiovascular events were designated as events requiring hospitalization or emergency room visit because of cardiovascular disease. ESA resistance to erythropoietin stimulating agents was defined as failure to achieve target hemoglobin levels (11–12 g/dL) with an epoetin dose < 300 IU/kg/week or a darbopoietin-α dose of 1.5 μg/week [23]. Non-dipper was defined as blood pressure decline of < 10% at night compared to that during the day [24].

### Measurement of residual renal function

Blood was sampled at the end of the first dialysis session of the week (blood urea 1) and immediately before the next session (blood urea 2). Between these blood samples, urine was collected throughout the interdialytic period. Residual renal urea clearance (KRU) was calculated using the following formula [25]:

$$Urea\;clearance\;(mL/\text{min})\;=\frac{2\;\times \;(urine\;urea\;concentration\;\times \;urine\;volume)}{urine\;collection\;duration\;\times \;(blood\;urea\;1\;+\;blood\;urea\;2)}$$

### Abdominal aortic calcification score

A lateral lumbar X-ray of the abdominal aorta was used to determine abdominal aortic calcification score (AACS) as described by Kauppila et al [9]. A lateral radiograph of the abdomen was performed in a standing position and the aorta was identified as the tubular structure coursing in front of the anterior surface of the spine. We used a semi-quantitative scoring system; only the abdominal aorta segments in front of the first to fourth lumbar vertebrae were considered. Points were assigned on a 0–3 scale to areas of calcification identified along the anterior or posterior surface of the aorta (0, absent; 1, small; 2, moderate; 3, large) according to the length of each calcified plaque with respect to the craniocaudal length of the closet vertebra. All radiographs were read by two investigators and consensus was reached on the interpretation of all films.

### Assessment of pulse wave velocity

Brachial-ankle PWV (baPWV) was measured using a Vascular profiler 1000 (VP-1000; Colin Co. Ltd., Komaki, Japan). Brachial and post-tibia arterial pressure waveforms were stored for 10 seconds by using extremity cuffs connected to a plethysmographic sensor and an oscillometric pressure sensor wrapped around the arm and ankle. The baPWV was automatically calculated from the distance between two arterial recording sites divided by transit time. The upper limits of baPWV were 1394/1264 cm/s, 1435/1361 cm/s, 1552/1433 cm/s, 1597/1609 cm/s and 1798/1915 cm/s for healthy male/female at 10 years interval (age range 20–70) [26].

### Measurement of ambulatory blood pressure monitoring

Twenty-four hour blood pressure monitoring was performed using Model P6 pressurometer device (Del Mar ReynoldsMedical Ltd., Hertford, United Kingdom) and the results were assessed using its computer software. An ocillometric cuff was attached to the upper arm without vascular access for hemodialysis. Patients were requested to have a rest for 10 minutes before the measurements. The Operations were performed according to the strict instruction. The measurements were performed at 15 minutes intervals during the day (06:00–24:00) and 30 minutes intervals at night (0:00–6:00).

### Echocardiographic measurements

Comprehensive echocardiographic measurements were performed using an ultrasound machine (Vivid 7; GE Vingmed Utrasound AS, Horten, Norway) with a 2.5 MHz probe, based on the imaging protocol in the American Society of Echocardiography guideline [27]. Left ventricular ejection fraction (LVEF) was estimated using the modified biplane Simpson’s method in apical two and four-chamber views. LV mass was determined using the method described by Devereus et al. and LV mass index (LVMI) was calculated by dividing the LV mass by the body surface area. Mitral inflow was assessed with Doppler echocardiography from the apical four-chamber view. The mitral inflow profiles were used to measure the peak mitral inflow velocities at the early (E), late (A) diastole, and its deceleration time (DT). Doppler tissue imaging of the mitral annulus was also obtained. From the apical four-chamber view, the early (E′) and late (A′) diastolic peak velocities were evaluated. Moderate to severe diastolic dysfunction was defined as E/E′ > 15 [28].

### Statistical analyses

Statistical analyses were performed using SPSS 19.0 (SPSS Inc., Chicago, IL, USA). The Kolmogorov-Smirnov test was used to test the normality of continuous variables. The normally distributed variables were expressed as mean ± SD and compared with the student’s t-test for two groups. The non-normally distributed variables were expressed as median and interquartile range and compared with Mann-Whitney U test for two groups. The categorical variables were expressed by frequencies and percentages and compared with either the chi-square test or Fisher’s exact test. The factors associated with AACS and baPWV were ascertained by multivariate regression analyses, which included age, the underlying cause of ESRD, duration on dialysis, mean interdialytic weight gain, and KRU. AACS, Ca × P, CRP, KRU, and parathyroid hormone, being non-normally distributed, were log-transformed before inclusion into the model. The cumulative incidence of cardiovascular events was calculated using the Kaplan-Meier product estimation method.

## Results

### Baseline characteristics according to residual renal urea clearance

The baseline characteristics of all patients and those when the patients were subsequently divided according to median KRU (0.9 mL/min/1.73m^2^) are shown in Table 1. The mean age was 59.1 ± 11.0 years and 55 (51.9%) were male. The median dialysis duration was 28.4 (interquartile range, 10.9–49.2) months. The underlying cause of ESRD was diabetes in 60 patients (56.6%). Compared to patients with a KRU ≥ 0.9 ml/min/1.73m^2^, those with a KRU < 0.9 ml/min/1.73m^2^ had a longer hemodialysis duration, larger mean interdialytic weight gain, and greater likelihood of taking calcium-based phosphate binders and diuretics, and resistance to ESA. In addition, the C-reactive protein (CRP) and β2-microglobulin levels were higher in patients with a KRU < 0.9 mL/min/1.73m^2^. Furthermore, AACS (4.0 [1.0–10.0] vs. 3.0 [0.0–8.0], P = 0.05) and baPWV (1836.1 ± 250.4 vs. 1676.8 ± 311.0 cm/s, *p* = 0.01) were significantly higher in patients with a KRU < 0.9 mL/min/1.73m^2^ than patients with a KRU ≥ 0.9 ml/min/1.73m^2^.

**Table 1:** Demographics, clinical characteristics, and biochemical variables in hemodialysis patients according to KRU.

Variables	Total (n = 106)	KRU < 0.9 mL/min/1.73m^2^ (n = 53)	KRU ≥ 0.9mL/min/1.73m^2^ (n = 53)	*p*-value
Demographic data				
Age (years)	59.1 ± 11.0	58.2 ± 9.1	60 ± 12.7	0.39
Male, n (%)	55 (51.9)	26 (49.1)	29 (54.7)	0.70
Clinical data				
Duration of hemodialysis (months)	28.4 (10.9–49.2)	37.4 (19.5–56.6)	13.6 (7.0–42.9)	<0.001
Initial end-stage renal disease				0.70
Diabetes, n (%)	60 (56.6)	31 (58.5)	29 (54.7)	
Non-diabetes, n (%)	46 (43.4)	22 (41.5)	24 (45.3)	
Previous cardiovascular disease				
Coronary artery disease, n (%)	43 (40.6)	23 (43.4)	20 (37.0)	0.99
Peripheral artery disease, n (%)	17 (16.0)	7 (13.2)	10 (18.9)	0.43
Cerebrovascular disease, n (%)	2 (1.9)	1 (1.9)	1 (1.9)	0.99
Mean interdialytic weight gain (kg)	1.5 ± 1.2	1.9 ± 1.3	1.1 ± 1.0	< 0.001
Residual renal urine (cc)	600 (200–1000)	250 (120–400)	1000 (800–1575)	<0.001
Medication use				
Vitamin D analogs, n (%)	55 (51.9)	27 (50.9)	28 (52.8)	0.76
Calcium-based phosphate binder, n (%)	45 (42.5)	30 (56.6)	15 (28.3)	0.01
Non calcium-based phosphate binder, n (%)	12 (11.3)	8 (15.1)	4 (8.0)	0.42
Diuretics, n (%)	74 (69.8)	32 (60.4)	42 (79.2)	0.03
ACEi or ARB, n (%)	74 (69.8)	36 (67.9)	38 (71.7)	0.67
ESA, n (%)	90 (84.9)	46 (86.8)	44 (83.0)	0.85
Resistance, n (%)	11 (10.4)	8 (15.1)	2 (3.8)	0.05
Laboratory data				
Hemoglobin (g/dL)	10.1 ± 1.2	10.1 ± 1.1	10.2 ± 1.3	0.85
Albumin (g/dL)	3.8 ± 0.5	3.8 ± 0.4	3.7 ± 0.5	0.86
Calcium (mg/dL)	8.7 (8.1–9.1)	8.7 (8.1–9.1)	8.6 (8.2–8.9)	0.92
Phosphate (mg/dL)	4.5 ± 1.2	4.6 ± 1.4	4.4 ± 1.1	0.51
Ca × P (mg^2^/dL^2^)	40.0 (31.9–44.4)	40.5 (34.2–45.1)	38.3 (30.8–43.8)	0.28
Cholesterol (mg/dL)	138 (107.5–163.5)	138 (107.0–160.0)	140.0 (110.5–164.5)	0.38
Triglyceride (mg/dL)	93.5 (65.0–147.3)	88.0 (57.0–129.0)	104.0 (78.5–158.0)	0.07
High-density lipoprotein (mg/dL)	41.0 (32.0–51.0)	41.0 (31.0–53.0)	41.0 (33.5–49.5)	0.83
Low-density lipoprotein (mg/dL)	78.0 (58.0–95.0)	72.0 (53.0–89.0)	86.0 (62.0–103.0)	0.02
CRP (mg/L)	0.6 (0.3–1.6)	0.9 (0.4–3.0)	0.5 (0.3–1.1)	0.03
Parathyroid hormone (pg/dL)	204.0 (105.7–398.6)	243.8 (102.4–415.2)	196.6 (118.8–346.0)	0.65
β2-microglobulin (mg/L)	19.9 ± 6.8	22.4 ± 6.7	17.3 ± 5.8	0.01
KRU (mL/min/1.73 m^2^)	0.9 (0.3–2.5)	0.3 (0.2–0.6)	2.5 (1.5–3.0)	<0.001
Single-pool Kt/V	1.6 ± 0.3	1.6 ± 0.4	1.5 ± 0.3	0.59
AACS	3.0 (1.0–8.0)	4.0 (1.0–10.0)	3.0 (0.0–8.0)	0.05
ABPM				
Daytime mean blood pressure	106.8 ± 13.5	108.9 ± 14.4	104.5 ± 12.4	0.09
Nighttime blood pressure	101.5 ± 14.9	102.6 ± 15.4	100.4 ± 14.3	0.47
Non-dipper, n (%)	79 (74.5)	41 (80.4)	38 (79.2)	0.99
baPWV (cm/s)	1756.5 ± 292.2	1836.1 ± 250.4	1676.8 ± 311.0	0.01

Note: values are expressed as median ± SD or median (interquartile range) or number (percentage).

Abbreviations: AACS, abdominal aortic calcification score; ABPM, ambulatory blood pressure monitoring; ACEi, angiotensin-converting enzyme inhibitor; ARB, angiotensin II receptor blocker; baPWV, brachial-ankle pulse wave velocity; Ca × P, calcium× phosphate; CRP, C-reactive protein; ESA, erythropoietinstimulating agent; KRU, residual renal urea clearance.

### Determining factors for abdominal aortic calcification score and Brachial-ankle PWV

On univariate analyses, log-AACS was significantly positively associated with age (ß = 0.38, *p* < 0.001), diabetes (ß = 0.25, *p* = 0.01), and mean interdialytic weight gain (ß = 0.27, *p* = 0.01). However, it was significantly negatively associated with log-KRU (ß = -0.32, *p* = 0.001). Log-KRU remained a significant negative correlation with log-AACS after adjusting age, the underlying cause of ESRD, duration on dialysis, and mean interdialytic weight gain (ß = -0.29, *p* = 0.002) (Table 2). In addition, on multivariate analysis, log-KRU remained also a significant negative correlation with baPWV (ß = -0.19, *p* = 0.05) (Table 3).

**Table 2:** Determining factors for abdominal calcification score in hemodialysis patients with residual renal function.

	Univariate model			Multivariate model		
	Beta	standard error	*p*-value	Beta	standard error	*p*-value
Age (y)	0.38	0.01	<0.001	0.39	0.01	<0.001
Male (vs. female)	0.16	0.19	0.09			
Initial end-stage renal disease (Diabetes vs. non-diabetes)	0.25	0.19	0.01	0.22	0.17	0.01
Log-duration of hemodialysis (months)	-0.05	0.41	0.64	0.01	0.35	0.99
Mean interdialytic weight gain (kg)	0.27	0.08	0.01	0.17	0.08	0.64
Log-KRU (mL/min/1.73m2)	-0.32	0.17	0.001	-0.29	0.16	0.002
Use of vitamin D analogs	-0.12	0.20	0.25			
Use of calcium-based phosphate binders	0.15	0.19	0.14			
Log-(Ca × P) (mg^2^/dL^2^)	0.02	0.49	0.81			
Log-CRP (mg/L)	0.07	0.18	0.53			
Log-Parathyroid hormone (pg/dL)	-0.11	0.23	0.30			
β2-Microglobulin (mg/L)	0.08	0.01	0.46			

Abbreviations: Ca × P; calcium × phosphate; CRP, C-reactive protein; KRU, residual renal urea clearance.

**Table 3:** Determining factors for brachial-ankle pulse wave velocity in hemodialysis patients with residual renal function.

	Univariate model			Multivariate model		
	Beta	standard error	*p*-value	Beta	standard error	*p*-value
Age (y)	-0.01	2.61	0.98	0.01	2.56	0.94
Male (vs. female)	0.05	56.99	0.61			
Initial end-stage renal disease (Diabetes vs. non-diabetes)	0.39	53.00	<0.001	0.38	54.14	<0.001
Log-duration of hemodialysis (months)	-0.09	120.78	0.38	-0.10	113.61	0.28
Mean interdialytic weight gain (kg)	0.20	24.80	0.05	0.13	24.44	0.18
Log-KRU (mL/min/1.73m2)	-0.25	50.21	0.01	-0.19	52.24	0.05
Use of vitamin D analogs	0.11	58.86	0.27			
Use of calcium-based phosphate binders	0.16	58.12	0.10			
Log-(Ca × P) (mg^2^/dL^2^)	-0.16	150.38	0.12			
Log-CRP (mg/L)	-0.16	54.92	0.11			
Log-Parathyroid hormone (pg/dL)	0.02	71.36	0.82			
β2-Microglobulin (mg/L)	0.08	4.38	0.42			

Abbreviations: Ca × P; Calcium × Phosphate; CRP, C-reactive protein; KRU, residual renal urea clearance.

### Echocardiographic parameters according to residual renal urea clearance

Table 4 presents the echocardiographic parameters of the two groups. The proportion of moderate to severe left ventricular diastolic dysfunction (67.9% vs. 49.1%, *p* = 0.05) were significantly higher in patients with a KRU < 0.9 mL/min/1.73m^2^ than in those with a KRU ≥ 0.9 mL/min/1.73m^2^. In addition, on multivariate regression analysis, log-KRU remained also a significant negative correlation with the E/E' ratio of echocardiac parameters (S1 Table). Of note, AACS (5.0 [1.0–9.0] vs. 2.0 [0.0–6.0], *p* = 0.01) and baPWV (1825.6 ± 306.9 vs. 1659.0 ± 241.2 cm/s, *p* = 0.003) were significantly higher in patients with moderate to severe left ventricular diastolic dysfunction than patients without that.

**Table 4:** Echocardiac parameters in hemodialysis patients according to KRU.

Variable	Total (n = 106)	KRU < 0.9 mL/min/1.73m2 (n = 53)	KRU ≥ 0.9 mL/min/1.73m2 (n = 53)	*p*-value
Echocardiac parameter				
LVEF (%)	57.2 ± 10.7	56.1 ± 11.9	58.4 ± 9.1	0.29
LVMI (g/m^2^)	139.8 ± 41.3	135.7 ± 42.3	144.2 ± 40.3	0.32
E (cm/s)	78.5 ± 24.0	75.8 ± 23.0	81.4 ± 25.0	0.26
A (cm/s)	91.4 ± 21.1	87.3 ± 18.8	95.8 ± 22.8	0.06
E/A ratio	0.9 ± 0.4	0.9 ± 0.4	0.8 ± 0.4	0.61
E’/A' ratio	0.6 ± 0.2	0.6 ± 0.2	0.6 ± 0.2	0.77
E/E' ratio	18.9 ± 9.1	20.6 ± 9.4	17.2 ± 8.6	0.06
Moderate to severe diastolic dysfunction, n (%)	62 (58.5)	36 (67.9)	26 (49.1)	0.05
DT (ms)	216.3 ± 67.9	213.9 ± 70.7	218.9 ± 65.5	0.74

Note: values are expressed as median ± SD

Abbreviations: DT, deceleration time; LVEF, left ventricular ejection fraction; LVMI, left ventricular mass index.

### Cardiovascular events according to residual renal urea clearance

**Fig 1:**
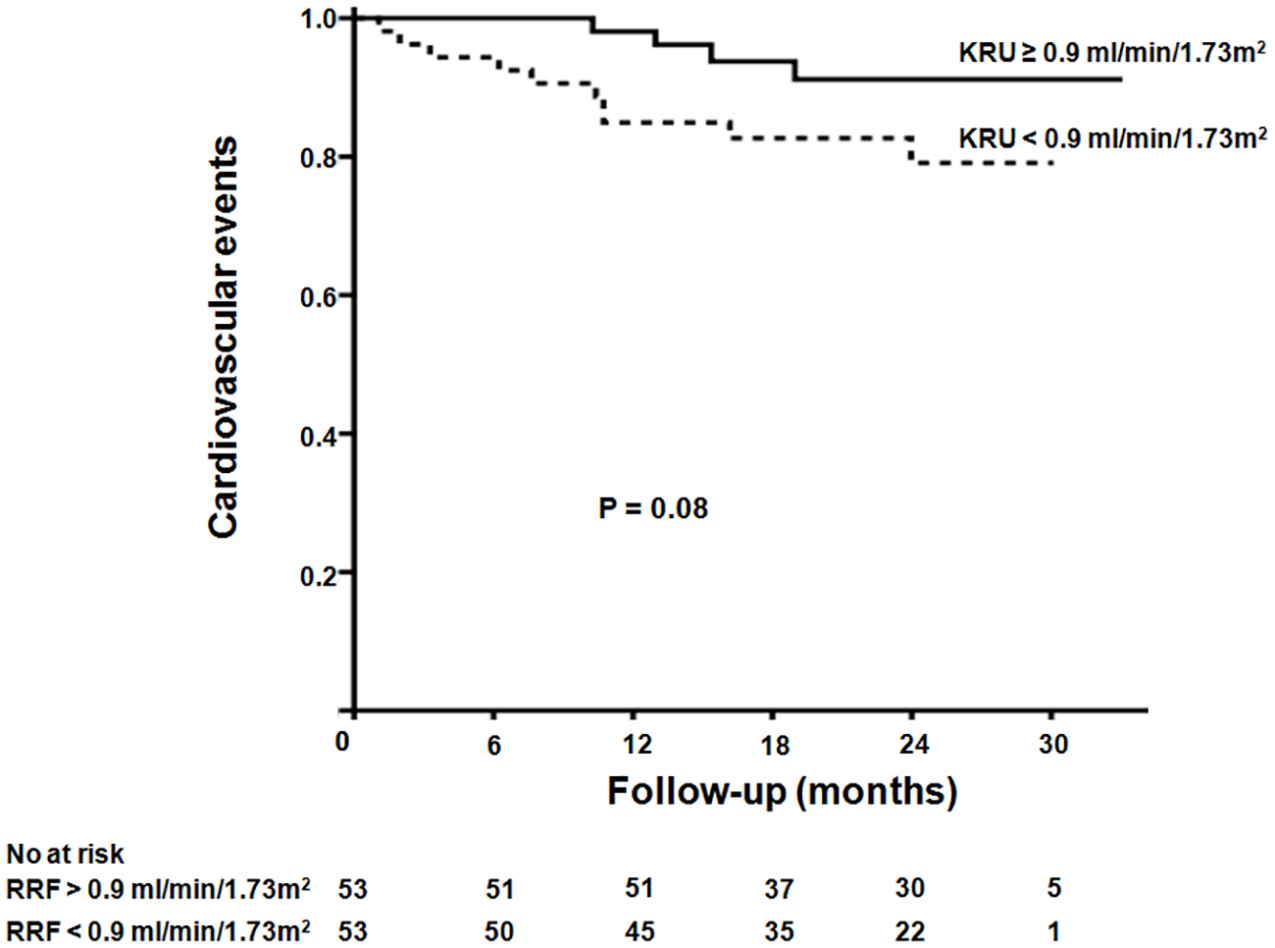
Kaplan-Meier analysis of cardiovascular events in hemodialysis patients according to KRU. Patients with a KRU < 0.9 mL/min/1.73m^2^ showed a higher tendency of cumulative cardiovascular events compared to those with a KRU ≥ 0.9 ml/min/1.73m^2^ (*p* = 0.08).

During a mean follow-up of 21.4 ± 7.1 months, 14 patients on HD (13.0%) experienced cardiovascular events. Of these patients, 10, three, and one experienced coronary artery disease, arrhythmia, and cerebrovascular disease, respectively. Cardiovascular events occurred in patients on HD with a KRU ≥ 0.9 mL/min/1.73m^2^ (four patients; 7.5%) and in patients on HD with a KRU < 0.9 mL/min/1.73m^2^ (10 patients; 18.9%). Although the Kaplan-Meier analysis revealed no significant intergroup difference, there is a trend that cumulative cardiovascular events were higher in patients with a KRU < 0.9 mL/min/1.73m^2^ than with a KRU ≥ 0.9 mL/min/1.73m^2^ (*p* = 0.08) (Fig 1).

## Discussion

This study showed that an increased AACS and baPWV were independently associated with deterioration of residual renal function. In addition, the proportion of left ventricular diastolic dysfunction was higher in patients with low residual renal function than in those with high residual renal function. Finally, Patients with low residual renal function showed a higher tendency of cumulative cardiovascular events compared to those with high residual renal function.

In our study, the proportion of patients taking calcium-based phosphate binders and with ESA resistance and β2-microglobulin and CRP levels were higher in patients with a KRU < 0.9 mL/min/1.73m^2^ than in those with a KRU ≥ 0.9 mL/min/1.73m^2^, which was consistent with other studies [18,21,29,30]. Although there had been lack of a standardized definition of residual renal function, the presence of residual renal function in patients on hemodialysis was defined as > 200 mL of urine/24 hours in many studies [21, 31–33]. However, in our study, patients on hemodialysis with the presence of urine were included regardless of urine volume. This may explain why median residual renal function expressed as GFR in our study was lower than those reported in other studies [21,29].

KRU is practically and widely used to measure residual renal function in dialysis. In patients on hemodialysis, residual renal function may vary over the dialysis cycle; thus, urine collection is required during the interdialytic period, usually 44 hours or 2 days to accurately estimate residual renal function. However, this can be difficult and inconvenient for patients on hemodialysis. In addition, the common belief that renal function rapidly declines after the initiation of hemodialysis may underappreciate the importance of residual renal function in patients on hemodialysis. For these reasons, many studies of residual renal function have been performed in peritoneal dialysis patients. However, this study involved urine collection during the interdialytic period to estimate residual renal function in patients on hemodialysis.

Vascular calcification is common in patients with ESRD. The severity of vascular calcification has been assessed using several methods [9,34]. Although cardiac computed tomography is a very accurate and reliable method for assessing cardiovascular calcification extent [34], it is expensive and involves radiation exposure. Therefore, the KDIGO consensus suggested the use of routine lateral lumbar radiography to measure AACS in clinical practice [35]. In addition, PWV and non-dipper pattern of ABPM is related with vascular stiffness. Although carotid-femoral PWV(cfPWV) is the current gold- standard method for assessing central arterial stiffness [36], as of convenience and feasibility, a simpler method of measuring PWV called baPWV is widely used in clinical practice.

Recent studies suggested that the vascular calcification of CKD patients is not simple precipitate calcium-phosphorous products but is related with multiple factors in the pathological process [37]. Generally, β2-microglobulin levels increase when RRF deteriorates in HD patients. Although it is still unknown whether β2-microglobulin is only a uremic toxin marker or also an active player in vascular damage, preclinical and clinical studies have shown that it plays an active role in vascular calcification [38]. Therefore, retained β2-microglobulin seems to further contribute to the vascular calcification process in the uremic milieu. In addition, oxidative stress and inflammation are also considered to play a key role [5]. Interestingly, Won et al showed that ESA resistance was associated with vascular calcification, which could be explained based on oxidative stress and inflammation [5]. Meanwhile, Stompor et al. described that higher residual renal function in dialysis patients was responsible for better clearance of proinflammatory cytokines [39]. Vascular calcification caused by these multiple factors is related to vascular stiffness. However, volume overload also increases vascular stiffness by vascular distension (Laplace’ law) [40]. Therefore, high interdialytic weight gain induced by deterioration of residual renal function is associated with increased vascular stiffness in patients on hemodialysis. Taken together, following the decrease in residual renal function of dialysis patients, the accumulation of proinflammatory cytokines and β2-microglobulin, and high interdialytic weight gain may induce the increase of vascular calcification and stiffness. Likewise, in the present study, we clearly showed that although AACS was not associated with the calcium-phosphorous products, it was significantly negatively correlated with KRU. In addition, baPWV was also significantly negatively correlated with KRU. However, because the proportion of non-dipper pattern was high in this study, there was no difference in non-dipper pattern according to KRU.

Vascular calcification and stiffness reduce aortic and arterial elastance, which impairs cardiovascular hemodynamics in patients with ESRD. In particular, aortic calcification cause left ventricular diastolic dysfunction by eroding compliance and elasticity [41]. In line with this finding, in this study, patients with moderate to severe left ventricular diastolic dysfunction showed higher AACS and baPWV than those without moderate to severe left ventricular diastolic dysfunction. In addition, the E/E' ratio of echocardiac parameters were independently associated with deterioration of residual renal function in this study. Of note, left ventricular diastolic dysfunction is known as an ongoing cardiovascular challenge and may be an independent predictor of cardiovascular events in dialysis patients [41]. In summary, the association of residual renal function with cardiovascular disease can be explained by more accumulated cytokines and uremic toxin and greater interdialytic weight gain given the likely more rapid loss of residual renal function. Although there was no significant difference in cardiovascular events in all patients with low residual renal function compared to those with high residual renal function, considering the short-term follow-up period of this study, a long observation period is required.

Our study had several limitations. First, the cause and effect relationship of residual renal function, vascular calcification, and left ventricular diastolic dysfunction cannot be determined using the cross-sectional study design. Second, the number of patients was small and follow-up period of this study was short to confirm whether residual renal function was an independent factor affecting cardiovascular events. Third, there was the possibility of inaccurate urine collection during the interdialytic period for measuring KRU. In addition, KRU measures only small molecule solute clearance, whereas residual renal function also contributes to convective clearance of mid-sized molecules. Therefore, KRU may not represent residual renal function. Fourth, because baPWV may be influenced by peripheral stiffness, cfPWV is a golden-standard method to measure aortic stiffness rather than peripheral baPWV.

In conclusion, although patients with high residual renal function had so much shorter hemodialysis duration before study enrollment in this study, it showed that increased AACS and baPWV were independently associated with deterioration of residual renal function after factor adjustment including hemodialysis duration. In addition, we founded that residual renal function was also independent factor for left ventricular diastolic dysfunction predicting cardiovascular events. However, these results also make it difficult to confirm the causal relationship between cardiovascular events and residual renal function. To confirm it, the analysis of time-to-event must be needed following the data is accumulated.

## Supporting information

S1 Table Determining factors for the E/E' ratio among echocardiac parameters in hemodialysis patients with residual renal function. (DOC)

S1 File Participant-level data. (XLS)
