# Supplementary material for: Vascular calcification and cardiac function according to residual renal function in patients on hemodialysis with urination
Source: PLoS One. 2017 Sep 27;12(9):e0185296. doi: 10.1371/journal.pone.0185296 (PMC5617191; doi:10.1371/journal.pone.0185296)
Supplement: S1 Table — (DOC) [file pone.0185296.s001.doc]

S1 Table. Determining factors for the E/E' ratio among echocardiac parameters in hemodialysis patients with residual renal function

|  |  | Univariate model | | |  | Multivariate model | | |
| --- | --- | --- | --- | --- | --- | --- | --- | --- |
|  |  | Beta | standard error | *p*-value |  | Beta | standard error | *p*-value |
| Age (y) |  | 0.09 | 0.08 | 0.37 |  |  |  |  |
| Male (vs. female) |  | -0.37 | 1.78 | 0.72 |  |  |  |  |
| Initial end-stage renal disease (Diabetes vs. non-diabetes) | | 0.19 | 1.76 | 0.05 |  | 0.22 | 1.70 | 0.02 |
| Log-duration of hemodialysis (months) |  | -0.16 | 3.73 | 0.10 |  | -0.17 | 3.56 | 0.08 |
| Mean interdialytic weight gain (kg) |  | 0.13 | 0.77 | 0.20 |  | 0.27 | 0.79 | 0.01 |
| Log-KRU (mL/min/1.73m2 ) |  | -0.27 | 1.56 | 0.01 |  | -0.33 | 1.86 | 0.004 |
| Use of vitamin D analogs |  | 0.05 | 1.78 | 0.62 |  |  |  |  |
| Use of calcium-based phosphate binders |  | -0.15 | 1.80 | 0.14 |  |  |  |  |
| Log-(Ca × P) (mg^2^/dL^2^) |  | -0.07 | 4.59 | 0.52 |  |  |  |  |
| Log-CRP (mg/L) |  | 0.13 | 1.67 | 0.21 |  |  |  |  |
| Log-Parathyroid hormone (pg/dL) |  | -0.56 | 2.15 | 0.56 |  |  |  |  |
| β2-Microglobulin (mg/L) |  | 0.22 | 0.13 | 0.03 |  | 0.07 | 0.14 | 0.51 |

Abbreviations: Ca Ⅹ P; Calcium Ⅹ Phosphate; CRP, C-reactive protein; KRU, residual renal urea clearance.
